# A Multi-Policy Rapidly-Exploring Random Tree Based Mobile Robot Controller for Safe Path Planning in Human-Shared Environments

**DOI:** 10.3390/s25227008

**Published:** 2025-11-17

**Authors:** Jian Mi, Xianbo Zhang, Zhongjie Long, Jun Wang

**Affiliations:** 1 Department of Transport Engineering, School of Civil Engineering and Transportation, Yangzhou University, Yangzhou 225127, China; jmi@yzu.edu.cn; 2 Key Laboratory of the Ministry of Education for Modern Measurement & Control Technology, College of Mechanical and Electrical Engineering, Beijing Information Science & Technology University, Beijing 102206, China; 2023020024@bistu.edu.cn; 3 College of Information Science & Technology, Beijing University of Chemical Technology, Beijing 100029, China; wangjunrob@buct.edu.cn

**Keywords:** mobile robot, safe path planning, human-shared environments, multi-policy rapidly exploring random tree search, stochastic risk evaluation

## Abstract

Mobile robot path planning in static environments has been extensively studied. However, ensuring a safe path in the presence of stochastically moving humans remains a significant challenge. This work focuses on solving the pathfinding problem of a mobile robot operating in human-shared environments with unknown human motions. To prevent conflicts at the planning level, we propose a multi-policy rapidly exploring random tree (MP-RRT)-based safe pathfinding algorithm. A MP-RRT diverse path generator is developed within this framework to produce multiple diverse candidate paths, which are considered as the initial solution set. Additionally, a dynamic quadrant-based stochastic exploration mechanism is introduced for efficient environment exploration. To obtain an optimally safe path, we design a path optimization mechanism based on stochastic risk evaluation, which explicitly models human motion uncertainties. Finally, an optimal safe path is generated by considering human risks at the planning level to ensure the safety for a robot collaborating with humans. We evaluate the proposed algorithm under different configurations ideal warehouse grid environment from conflict numbers, task success rate, and path reward. The proposed method outperforms A*, MDP, and RRT in terms of conflict number (−70.2%, −72.8%, and −73.8%), task success rate (+66.0%, +95.0%, and +85.7%). Simulation results prove the efficiency of our proposals in safe path planning in human-shared environments.

## 1. Introduction

In recent years, substantial advances in robotics technology have greatly accelerated the development of autonomous systems, enabling transformative applications across diverse real-world domains, including automated factories [1], logistics centers [2], warehouses [3], and manufacturing systems [4]. These advancements encompass not only autonomous mobile robots (AMRs) but also humanoid robots. The demand for robots capable of collaborating with humans has been steadily increasing [5,6,7,8]. As is well known, path planning is a fundamental and essential component of robot control [9,10]. Although path planning in static environments has been extensively researched [11,12], planning a safe path for a mobile robot operating alongside stochastically moving humans remains a challenging problem [13,14,15]. Resolving potential conflicts between robots and humans has become a critical and pressing challenge for effective human–robot collaboration.

Safety remains a primary bottleneck in human–robot collaboration [16] and various research has been conducted to address this issue [17,18,19,20]. In addition to safety, the task success rate constitutes a factor as critical as safety for effective human–robot collaboration in the industrial domain. Classic pathfinding algorithms, such as D* [21], A* [22], and RRT [23], are efficient in finding an optimal path without environment uncertainties. However, the objective of path planning in human–robot collaboration differs from the classical Shortest Path Planning (SPP) problem [24,25]. In this context, the planned path must ensure not only safety by avoiding potential future conflicts with humans but also efficiency by enabling timely arrival at the goal within a given time budget.

Numerous approaches have been proposed to address the dynamic obstacle avoidance problem [26,27,28,29,30]. Among them, real-time path planning algorithms [31,32] are widely used, allowing robots to continuously detect their environment, make decisions, and execute actions to avoid dynamic obstacles. This iterative process is currently recognized as one of the most reliable methods for ensuring safety as it replans the path when it encounters a dynamic obstacle [33]. Typically, this approach employs a global planner to guide the robot toward its goal and a local planner for obstacle avoidance [34]. However, real-time solution schemes focus primarily on avoiding immediately encountered conflicts and lack foresight, which can result in a loss of global optimality and a low task task success rate. In addition, such approaches often incur high computational costs due to continuous re-planning. In industrial domains, robots are deployed to perform tasks with the objective of improving overall work efficiency. Real-time solution schemes are not applicable in industrial scenarios where humans move stochastically. To the best of our knowledge, few warehouses or logistics centers currently allow mobile robots to operate directly alongside human staff without physical separation or safety barriers. The main reason is that the safety of a path cannot be guaranteed as future human motions are unknown. Once a real-time solution scheme is applied to ensure safety, the work efficiency tends to decrease.

In order to enable a robot to work in human-shared environments, various human-aware [35,36]- or risk-aware [37,38]-based path planning methods have been developed that rely on monitoring human movements. However, those methods rely heavily on detouring the encountered human. Our previous studies [14,15] have demonstrated that addressing conflicts at the planning level provides an effective solution for navigation in dynamic environments. Resolving future potential conflicts at the planning level significantly reduces computational costs compared with continuous re-planning at every time step. Based on the principle of solving conflicts at the planning level instead of re-planning when encountering humans, we propose a multi-policy rapidly exploring random tree (MP-RRT)-based mobile robot controller for safe path planning without prior knowledge of human motions. The motivation behind our proposal is to avoid stochastic conflicts with humans while maintaining a high task success rate in environments like warehouses where robots and humans work together. The key contributions of this study are summarized as follows:

1. We develop a novel MP-RRT-based safe path finding algorithm to generate an optimal safe path under unknown human motions.
2. An MP-RRT diverse path generator is proposed to generate multiple diverse path candidates which focus more on breadth first search (BFS).
3. A dynamic quadrant stochastic exploration mechanism is developed to enable multi-policy searching.
4. A safe path optimization mechanism based on stochastic risk evaluation is constructed to obtain an optimal safe path.

The rest of this paper is organized as follows. Section 2 presents a review of related works. Section 3 introduces the problem formulation. The proposed safe planner is presented in Section 4, followed by the simulation results in ideal warehouse grid environment and discussions in Section 5. Finally, we give a brief conclusion in Section 6.

## 2. Related Works

The path planning problem in dynamic environments aims to move a robot to its goal position without conflicts with environmental uncertainties. Classical path planning methods, such as A* [39,40], D* [21,41], and RRT/RRT* [42,43], have demonstrated their effectiveness in solving pathfinding problems in static environments. Numerous variants of these algorithms have been extended to address path planning in dynamic environments. We roughly classify the related methods into three types: searching-based methods, sampling-based methods, and learning-based methods. In the following, we provide a review of related methods.

Searching-based methods explore the environment by incrementally generating and evaluating successor states, primarily represented by variants of A*, D*, and related graph search algorithms. J. Mi et al. [15] developed a safe A* algorithm to plan a safe local path while considering stochastic human risks. However, their algorithm is limited and highly dependent on the reward design. M. Je Choi et al. [35] proposed an enhanced D* algorithm to optimize the path for an autonomous robot moving on sidewalks. They analyzed people’s trajectory data collected by lidar sensors, and identified the average distance and angle of avoidance at which people start to avoid autonomous robots. Their algorithm allows the robot to maintain its existing optimal path when humans are willing to maneuver around it. However, strictly speaking, this problem setting assumes that humans avoid robots rather than requiring robots to avoid humans.

Sampling-based methods explore the environment by drawing samples according to predefined policies, most commonly random sampling, to incrementally approximate the feasible space. V. Rajendran et al. [36] proposed a human-aware RRT-connect motion planner to maintain a safe distance. However, their setting involves human and manipulator collaboration, not a robot working with randomly moving humans. Jyotish and M. Chen [43] presented a TD-RRT* dynamic re-planning method to avoid both moving and static obstacles. However, their obstacle avoidance mechanism depends on real-time re-planning once the path is obstructed.

For learning-based methods, reinforcement learning (RL) [13,44] and Monte Carlo Tree Search (MCTS) [45,46] are two representative approaches. These methods typically optimize an objective function by designing reward mechanisms and assigning penalties when conflicts occur. Chen et al. [47] introduced a safety-oriented RL controller that integrates control barrier functions, in which Gaussian processes were utilized to model system dynamics and associated uncertainties. However, Gaussian kernels often struggle to represent multi-modal uncertainty distributions. El-Shamouty et al. [16] and Liu et al. [48] applied deep reinforcement learning techniques to achieve safe motion planning in human–robot collaboration scenarios. Luo et al. [49] proposed AlphaRoute, an AlphaGo-inspired planner for large-scale route coordination based on graph attention reinforcement learning and MCTS. Learning-based path planning methods have demonstrated strong adaptability in dynamic environments. However, their training process is computationally expensive, and these methods still lack theoretical guarantees in terms of safety and optimality.

The approaches to solving human and robot conflicts can also be divided into three types: real-time detection, simulation, and cost-map construction. Works like [43,50] use the real-time detection method for obstacle avoidance, which represents one of the most common solutions. It is the safest way and is widely applied in robotics, self-driving, and other fields. As described previously, it incurs heavy computational cost and may lead to a low task success rate. Both RL and MCTS [51] penalize the robot by simulations with designing reward functions that are simple and efficient. However, it cannot provide any theoretical guarantee. L. Bruckschen et al. [52] compute a time-dependent cost map to realize human-aware robot navigation. It also involves expensive computational cost and requires large memory capacity. A. Dixit et al. [53] applied conditional value at risk (CVaR) to calculate risk constraints, which has shown promising results in handling environmental risks. Unfortunately, CVaR is hard to estimate.

Although the aforementioned methods have achieved significant progress in path planning for dynamic environments, several limitations prevent their deployment in industrial applications Searching-based methods exhibit limited adaptability to environmental changes, and sampling-based approaches suffer from the same drawback. Learning-based algorithms provide a promising framework for handling uncertainties; however, their high computational cost restricts practical use in industrial settings. Moreover, the paths generated by these methods are often not robust to future environment changes. Most existing approaches rely on online replanning when encountering moving obstacles, which easily traps the planner in local optima and reduces task success rates, especially when the robot operates under strict time constraints. Therefore, resolving potential conflicts proactively at the planning level is crucial to improving the efficiency and safety of human–robot collaboration in industrial environments.

In this paper, we simulate the human trajectories and calculate the risk distributions which is efficient and more simple than CVaR. Unlike traditional human-aware methods, we try to generate multiple diverse paths distributed over the whole environment to find an optimal safe path, increasing the path robustness and ensuring the safety of the robot and humans. A major difference from well-known online replanning approaches in human-shared environments is that we propose a safe path planning method solving conflicts at the planning level and find a safe path in a single planning process, that is, planning only one time. Hence, our method eliminates the expensive replanning cost for online replanning.

## 3. Problem Formulation

A mobile robot collaborates with *K* $\left(K\in N_{+}\right)$ humans. Note that the movements of each human are unknown. A human moves according to their own decisions and cannot be controlled. We model the environment as follows. Let

- *S* be the finite state set of the robot, $s_{t}\in S$ define the state of the robot at discrete time-step *t*, and $s_{0}$, $s_{g}$ be the initial state and goal state, respectively,
- *H* be the finite state set of humans, $h_{t}=\left\{h_{t}^{1},\ldots ,h_{t}^{K}\right\}\in H$ define the state of *K* humans at time-step *t*, and $h_{0}^{k}$, $h_{g}^{k}$ be the initial state, goal state of human *k*, respectively,
- A={←,→,↑,↓,wait} represent the action set of the robot and humans.

Let $x=\{s_{0},\ldots ,s_{t},\ldots ,s_{T}\}$ be the path of the agent and *T* be the budget, $s_{T}=s_{g}$. For each human *k*, $h_{t}^{k}$ can be observed, and $h_{0}^{k}$, $h_{g}^{k}$ are known. However, the trajectory is unknown, and each human moves stochastically. The robot has to move to its goal $s_{g}$ without colliding with humans. A conflict occurs once the robot and a human *k* occupy the same position or traverse the same edge, that is,

- a vertex conflict occurs when $s_{t}=h_{t}^{k}$, and 
- an edge conflict occurs when $s_{t}=h_{t+1}^{k}\wedge s_{t+1}=h_{t}^{k}$.

The robot achieves a success if and only if it reaches the goal without collisions and within the budget *T*. Otherwise, it suffers a failure.

We aim to find an optimal safe path for the robot with unknown human motions. As described above, we can only observe the state of a human *k*, $h_{t_{0}}^{k}$, at time step $t_{0}$, and the destination $h_{g}^{k}$ is also known. However, the most critical knowledge—the paths of humans—remains unknown. We do not have prior information about their movements. The problem we aim to address is how to avoid stochastic conflicts between the robot and randomly moving humans. In this paper, we focus on the safety of the generated path rather than on achieving the traditional shortest path.

## 4. MP-RRT-Based Mobile Robot Controller

**Figure 1:**
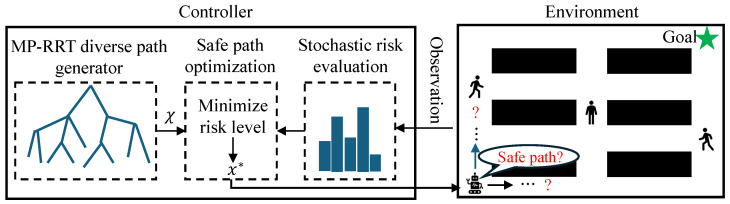
Architecture of the proposed controller. $x^{*}$ is the optimal path. The arrows indicate the direction of data or process flow (left) and the move direction of the robot (right). Green star is the goal position of the robot. Black rectangles are static obstacles.

The architecture of the proposed mobile robot controller is illustrated in Figure 1. The controller consists of a stochastic risk evaluation module, an MP-RRT diverse path generator, and a safe path optimization module. Hereafter, we present the details of each module.

### 4.1. Model Human Uncertainties: Stochastic Risk Evaluation

**Figure 2:**
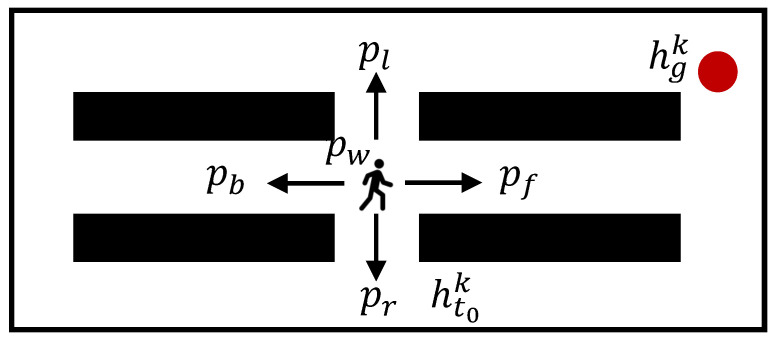
Stochastic motion model of the human. A human moves to his/her goal or take a “wait” action with a stochastic policy. Red circle is the goal of human *k*.

It is difficult to obtain the precise movements of humans. We perform stochastic simulations to model the risk caused by humans. Figure 2 shows the stochastic motion model of a human *k*. We know that a human *k* will go to his/her goal location. However, the path that a human takes is unknown. We model the human motions as follows. The probability of each action *a* (a∈A) is modeled as $p_{a}\in \left\{p_{f},p_{b},p_{l},p_{r},p_{w}\right\}$ with respect to the action set *A*. Note that $p_{a}$ is unknown.

All humans move according to the following policies:

- each human prefers movements directed toward their goal;
- no conflicts occur between any two humans, that is, for any two humans $k_{1}$, $k_{2}$,
- $h_{t}^{k_{1}}=h_{t}^{k_{2}}$ and $h_{t}^{k_{1}}=h_{t+1}^{k_{2}}\wedge h_{t+1}^{k_{1}}=h_{t}^{k_{2}}$, are not allowed.

For any time step *t*, a human *k* takes an action $a_{t}$ at $h_{t}^{k}$, and the state transition is written as $\left(h_{t}^{k},a_{t},h_{t+1}^{k}\right)$. Let $A(h_{t}^{k})$ define the action set of human *k* at *t* and $A(h_{t}^{k})\subseteq A$. As described previously, a human prefers movements directed toward the goal. Let $a^{*}$ be the action that brings the robot closest to the goal and defined by

(1) $$a^{*}\leftarrow \arg\min_{a\in A(h_{t}^{k})}Manh\left(h_{t+1}^{k},h_{g}^{k}\right),$$

where Manh(·) calculates the manhattan distance from $h_{t+1}^{k}$ to $h_{g}^{k}$.

Let *C* be a set of prioritized actions, $C\subseteq A(h_{t}^{k})$, and let it contains all possible $a^{*}$. Higher conditional probabilities are assigned to prioritized actions to encourage goal-directed movements. Based on this principle, the actions are sampled from the following conditional probability distribution:

(2) $$p\left(a_{t}|h_{t}^{k}\right)=\left\{\begin{array}{cc} \frac{1-\zeta \left(|A(h_{t}^{k})|-|C|\right)}{\left|C\right|}, & a_{t}\in C,\\ \;\zeta , & \mathrm{otherwise}, \end{array}\right.$$

where |·| is the size of action set, 0≤ζ<0.2, $\sum_{a_{t}\in A\left(h_{t}^{k}\right)}p\left(a_{t}|h_{t}^{k}\right)=1$.

In a 2D grid environment, $h_{t_{0}}^{k}=v$ is the observed state at $t_{0}$, where the vertex *v* is occupied by the human *k*. We conduct stochastic simulations to predict the movements of the human from the observation $h_{t_{0}}^{k}$. This allows us to calculate the conditional probability of a vertex *v* occupied by human *k* at time step t (0<t≤T) as follows:

(3) $$p_{k,t}\left(v|h_{0}^{k}\right)=\frac{N_{v}}{N_{s}},$$

where $N_{s}$ represents the total simulation times and $N_{v}$ indicates the visited times of *v* at *t*. Then, the risk value of a vertex *v* occupied by all *K* humans at *t*, written as R(v,t), is calculated by

(4) $$R\left(v,t\right)=\sum_{k=1}^{K}p_{k,t}\left(v|h_{0}^{k}\right).$$

Finally, we obtain human risk set $\xi =\{\xi_{t_{0}},\xi_{t_{0}+1},\ldots ,\xi_{t_{0}+T}\}$ and $\forall \xi_{t}\in \xi$, $\xi_{t}=\left\{R\left(v_{1},t\right),R\left(v_{2},t\right),\ldots \right\},\;t_{0}\leq t\leq t_{0}+T$. Algorithm 1 shows the procedures of human risk estimation.
**Algorithm 1:** Human stochastic risk evaluation
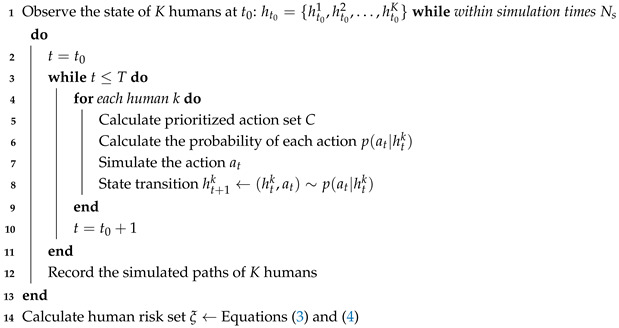


### 4.2. Multi-Policy Rapid-Exploring Random Tree (MP-RRT) Diverse Path Generator

The core part of the MP-RRT diverse path generator is the MP-RRT diverse path planning algorithm. It searches multiple paths with different policies while considering the diversity of paths.

#### 4.2.1. Multi-Policy Sampling

Unlike the traditional RRT algorithm, our method aims to generate multiple paths while accounting for path diversity. To this end, we design a multi-policy sampling strategy to explore the environment, which includes dynamic quadrant, bridge, and goal policies.

**Figure 3:**
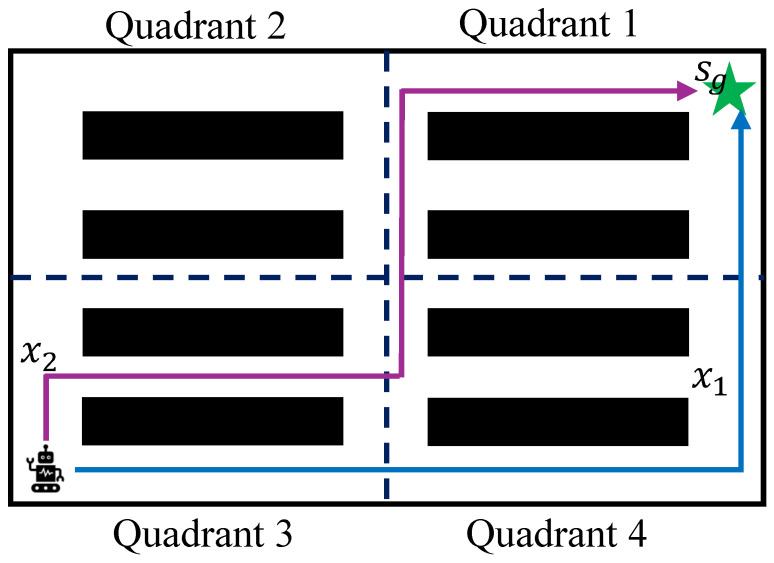
Quadrant exploration. The environment is divided into four quadrant areas by the black dashed lines. $x_{1}$ and $x_{2}$ are two paths across different quadrant areas.

**Dynamic quadrant policy (*****DQP*****).** Given a map G=<V,E>, as shown in Figure 3, the robot divides it into quadrants 1, 2, 3 and 4. Let $V_{1}$ be the vertex set which contains all vertices in quadrant 1. Similarly, we define the vertex set of quadrant 2, 3, 4 as $V_{2},V_{3},V_{4}$, respectively. Redefine $V=\{V_{1},V_{2},V_{3},V_{4}\}$ and $V=V_{1}\cup V_{2}\cup V_{3}\cup V_{4}$. $\forall V_{i}\in V$, i∈{1,2,3,4}, let ${\bar{V}}_{i}$ contain those vertices of quadrant *i* which has already been explored and are on one generated path *x*. For a vertex *v*, if $v\in V_{i}$ and v∈x, we have $v\in {\bar{V}}_{i}$. For each quadrant *i*, we define a exploration rate $\Delta (V_{i})$ by

(5) $$\Delta \left(V_{i}\right)=\frac{|{\bar{V}}_{i}|}{|V_{i}|},$$

where |·| is the size of a set.

Let χ be the set of robot paths. At the beginning, the environment has not been explored, χ=∅, ${\bar{V}}_{i}=\emptyset$ and $|{\bar{V}}_{i}|=0$, thus, the exploration rate $\Delta (V_{i})=0$.

(6) $$\chi =\emptyset \Rightarrow \forall V_{i}\subset V,\left|V_{i}\right|=0,\Delta \left(V_{i}\right)=0.$$

Once one or more paths are found, $\chi =\{x_{1},\ldots \}$, and quadrant *i* is explored, its exploration rate $0<\Delta (V_{i})$.

(7) $$|\chi |>0\Rightarrow \exists V_{i}\subset V,\;\left|{\bar{V}}_{i}\right|>0,\;\Delta \left(V_{i}\right)>0.$$

If quadrant *i* is fully explored, that is, all vertices of quadrant *i* have been added to $\bar{V_{i}}$, ${\bar{V}}_{i}=V_{i}$ and the exploration rate $\Delta (V_{i})=1$.

(8) $$|\chi |>0,\;V_{i}={\bar{V}}_{i}\Rightarrow \Delta \left(V_{i}\right)=1.$$

Let $\alpha_{1},\alpha_{2},\alpha_{3}$ and $\alpha_{4}$ be the sampling rate of quadrant 1, 2, 3, and 4. For each quadrant *i*, the sampling rate $\alpha_{i}$ changes along the exploration rate $\Delta (V_{i})$ and is calculated by

(9) $$\alpha_{i}==\frac{1-\Delta (V_{i})}{4-\Delta \left(V_{1}\right)-\Delta \left(V_{2}\right)-\Delta \left(V_{3}\right)-\Delta \left(V_{4}\right)}.$$

At the beginning, $\Delta \left(V_{1}\right)=\Delta \left(V_{2}\right)=\Delta \left(V_{3}\right)=\Delta \left(V_{4}\right)=0$, the sampling rate of each quadrant *i* follows the uniform distribution where $\alpha_{i}=0.25$. Once quadrant *i* is fully explored, $\Delta (V_{i})=1$, its sampling rate $\alpha_{i}=0$. The quadrant *i* won’t be explored any more. Let $\bar{\alpha }$ be the probability of the dynamic quadrant policy that the robot takes for sampling which is unknown.

**Bridge policy (*****BP*****).** This policy enables to sample a point from the search tree randomly. Hence, a new path could be found by bridging existing paths. The probability of bridge policy is β (0≤β<1).

**Goal policy (*****GP*****).** This policy is the same as the classic RRT/RRT* method. The proposed algorithm follows a probability of γ to sample the goal point (0≤γ<1).

#### 4.2.2. Diversity Evaluation

Given two paths $x_{1},x_{2}$, we evaluate the diversity of them using function $D(x_{1},x_{2})$. $D(x_{1},x_{2})$ is calculated based on Jaccard similarity,

(10) $$D\left(x_{1},x_{2}\right)=1-\frac{|x_{1}\cap x_{2}|}{|x_{1}\cup x_{2}|},$$

where $|x_{1}\cap x_{2}|$ is the the size of intersection set of $x_{1}$ and $x_{2}$ and $|x_{1}\cup x_{2}|$ is the size of the union set of two paths. The diversity increases when $D(x_{1},x_{2})$ varies from 0 to 1. $D(x_{1},x_{2})=0$ indicates that $x_{1}$ and $x_{2}$ are the same path while $D(x_{1},x_{2})=1$ shows two completely different paths.

Our purpose is to generate multiple paths while keeping their diversity. Let θ be the threshold of path diversity. For any two paths $x_{1},x_{2}\in \chi$, the following constraint is satisfied,

(11) $$D(x_{1},x_{2})\geq \theta .$$

#### 4.2.3. Searching Structure

**Figure 4:**
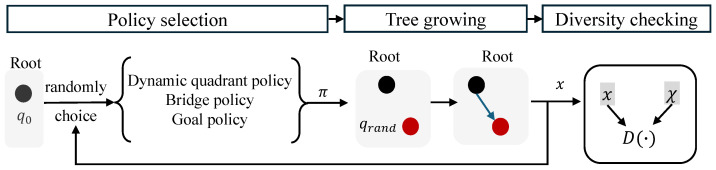
Searching structure of MP-RRT diverse path generator. redBlack node is the root and the red one is the $q_{rand}$. The blue arrow shows the growing direction.

The robot starts exploring the environment by sampling and growing the search tree. The searching structure of the proposed MP-RRT diverse path generator is shown as Figure 4. Let $\tau =<V_{\tau },E_{\tau }>$ be the search tree where $V_{\tau }$ is the node set and $E_{\tau }$ is the edge set. $q_{0}=s_{0}$ is the root node. Thus $V_{\tau }=\left\{q_{0}\right\}$ and $E_{\tau }=\emptyset$.

**Policy selection phase.** The robot starts sampling a point in the state space based on multiple policies–dynamic quadrant, bridge and goal. As described previously, the probability of each policy, $\bar{\alpha }$, β and γ are unknown. Hence, the robot randomly chooses a policy π from the above three policies as shown in Figure 4.

**Tree growing phase.** Based on the chosen policy π, a sampling point $q_{rand}$ is obtained. Then, $\forall q\in V_{\tau }$, we calculate the Manhattan distance from *q* to $q_{rand}$, written as $Manh(q,q_{rand})$. A nearest neighbor node $q_{m}$ with the minimum distance is chosen,

(12) $$q_{m}\leftarrow \arg min_{q\in V_{\tau }}Manh\left(q,q_{rand}\right).$$

Hence, the tree grows in the direction from $q_{m}$ to $q_{rand}$. As shown in Figure 4, $V_{\tau }$ contains only a root node. The growing direction is shown in blue arrow. The rest parts are similar to classic RRT method. We briefly introduce as follows. It moves from $q_{m}$ toward $q_{rand}$ and produces a new node $q^{\prime }$. If no obstacles exist between $q_{m}$ and $q^{\prime }$, add $q^{\prime }$ as the child of $q_{m}$; otherwise, discard it. Repeat sampling and growing the search tree until a path *x* is found or the computational budget runs out.

**Diversity checking phase.** For the new found path *x* and any existing path $x^{\prime }\left(x^{\prime }\in \chi \right)$, we check whether *x* satisfies the diversity constraints (Equation (11)) based on Equation (10). If $D(x,x^{\prime })\geq \theta$, add *x* to χ; otherwise, discard *x* and re-generate a new path until computational budget runs out or |χ| reaches the terminal condition, a desired path number *L*. The procedures are shown in Algorithm 2.

**Algorithm 2:** MP-RRT diverse path generator

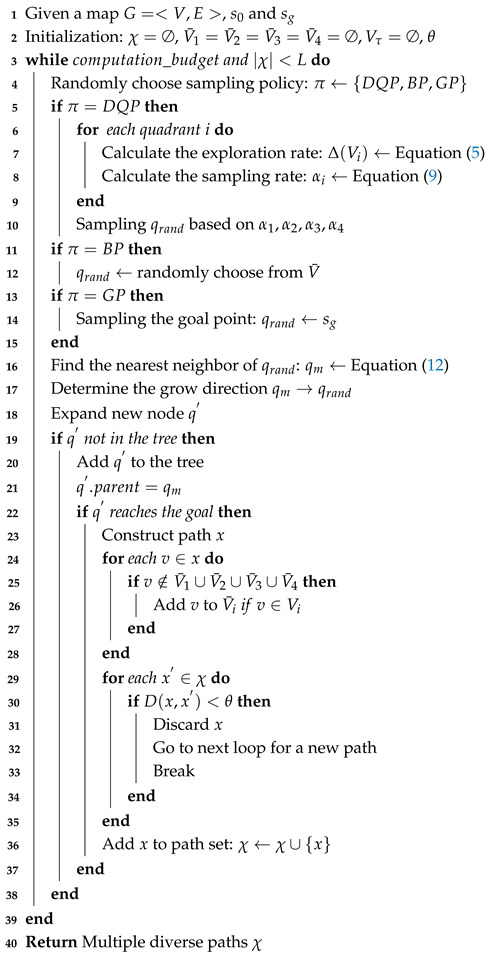



### 4.3. Safe Path Optimization

The MP-RRT diverse path generator outputs multiple diverse paths χ without considering unknown human motions. In this section, we show how an optimal safe path is generated. Each path *x* (x∈χ) is a candidate. We evaluate the conflict risk level of each candidate *x* based on the obtained human risks ξ.

For a candidate *x*, ∀v∈x, the probability of it being occupied by humans at time step *t* is R(v,t). Hence, the robot collides with a human at position *v* at *t* with a risk of R(v,t), which is the vertex conflict. As for the edge conflict, suppose that the robot moves to $v^{\prime }$ at time step t+1 and it encounters an edge conflict risk of $R\left(v,t+1\right)\times R\left(v^{\prime },t\right)$. The risk level of *x* is calculated by

(13) $$\begin{array}{c} Risk\left(x\right)=\sum_{t=0,v\in x}^{T}R\left(v,t\right)+\sum_{t=0,v,v^{\prime }\in x}^{T-1}\left(R\left(v,t+1\right)\times R\left(v^{\prime },t\right)\right). \end{array}$$

By minimizing the risk level, an optimal candidate path $x^{*}$ could be found, where

(14) $$x^{*}\leftarrow \arg min_{x\in X}Risk\left(x\right).$$

The procedures are shown as Algorithm 3.

**Algorithm 3:** Optimization

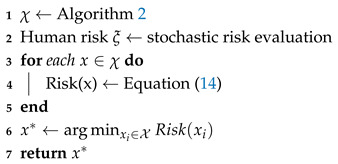



## 5. Results and Discussions

### 5.1. Environment Setting

To evaluate the effectiveness of the proposed methods, we conducted a series of scenario simulations and systematically compared the results with established algorithms such as A*, RRT and RL methods (MDP (Markov Decision Process), DQN (Deep Q-network), DDQN (Double Deep Q-network)). Note that we only perform simulations at the current stage and the experiments in real-world has not been performed. The simulations were performed on a high-performance computing system equipped with an Intel Core i9-10900 CPU running at 2.80 GHz and 32 GB of memory, ensuring sufficient computational resources for reliable assessment.

For all methods under evaluation, we adopted a consistent reward structure to maintain comparability for path quality evaluation. Specifically, the reward parameters were set as follows:

- goal achievement reward $r_{g}=10$,
- step penalty $r_{s}=-0.1$, which incentivizes shorter paths,
- conflict penalty $r_{c}=-2$, imposed when the agent encounters a collision with a human.

The robot receives a reward for the achievement of the goal $r_{g}$ within the given time step budget *T*. Throughout the navigation process, each step incurs a penalty of $r_{s}$, discouraging unnecessary movements and promoting efficiency. Additionally, to simulate the shared environment with humans, any collision with a human results in a penalty of $r_{c}$.

Parameters of multi-policy $\bar{\alpha },\;\beta ,\;\gamma$ are unknown. Table 1 presents the investigation results for several combinations of the parameters $\bar{\alpha }$, β, and γ. We find that the performance differences among these combinations are minor and it is difficult to determine the best parameter set. Therefore, a random number generator (RNG) is adopted to randomly assign the values of $\bar{\alpha }$, β, and γ under the following constraints:

- $\bar{\alpha }\in \left[0,1\right],\beta \in \left[0,1\right],\gamma \in \left[0,1\right]$, and
- $\bar{\alpha }+\beta +\gamma =1$.

**Table 1:** Performances of different parameter values $\bar{\alpha },\;\beta ,\;\gamma$.

Parameters	Average Conflict Number	Task Success Rate
$\bar{\alpha }=0.8$, β=0.1, γ=0.1	0.04 ± 0.20	0.96 ± 0.20
$\bar{\alpha }=0.7$, β=0.2, γ=0.1	0.05 ± 0.26	0.96 ± 0.20
$\bar{\alpha }=0.6$, β=0.3, γ=0.1	0.04 ± 0.20	0.96 ± 0.20
$\bar{\alpha }=0.5$, β=0.4, γ=0.1	0.05 ± 0.22	0.95 ± 0.22
$\bar{\alpha }=0.4$, β=0.5, γ=0.1	0.04 ± 0.20	0.96 ± 0.20
$\bar{\alpha }=0.4$, β=0.4, γ=0.2	0.06 ± 0.24	0.94 ± 0.24
$\bar{\alpha }=0.4$, β=0.3, γ=0.3	0.04 ± 0.20	0.96 ± 0.20
$\bar{\alpha }=0.3$, β=0.3, γ=0.4	0.06 ± 0.24	0.94 ± 0.24
$\bar{\alpha },\;\beta ,\;\gamma$: randomly generated	0.04 ± 0.20	0.96 ± 0.20

We verify the proposed methods in three scenarios:

- Scenario 1: verification on a small size map;
- Scenario 2: verification with different size maps;
- Scenario 3: verification on increasing human numbers.

**Figure 5:**
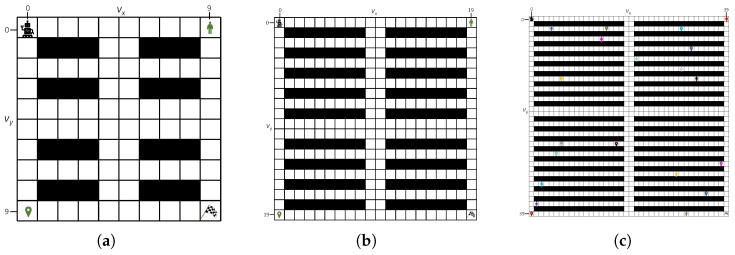
Warehouse grid environment map: (**a**) 10×10, (**b**) 20×20, and (**c**) 40×40. The flag indicates the goal of the robot and the map pin denotes the goal of a human. The start and goal positions of one human are represented in the same color. Black squares are obstacles.

Figure 5 shows the configuration of three different maps: 10×10, 20×20, and 40×40, which are warehouse grid environment. We run $N_{s}=2000$ times simulations to calculate human risks ξ. The diversity threshold θ is set as 0.25.

The evaluation metrics of different algorithms vary depending on the specific problem and scenario being addressed. For the shortest path planning problem [24,25], the primary metric is typically the path length. In the case of multi-agent path planning [54,55], commonly used metrics include the makespan or the sum of costs of all paths. Moreover, the task success rate and path reward are two widely adopted metrics in the literature for assessing overall performance and path quality.

In this paper, we focus more on the safety of the path. Therefore, the performance of the proposed method is evaluated using the following metrics: the average conflict number, conflict distribution, task success rate, and path reward. To ensure statistical reliability, each scenario was simulated 100 times, and the mean values of these metrics were calculated. A random seed set is applied for 100 simulations, [1000,1001,⋯,1099].

### 5.2. Verification on Scenario 1: Human Number K = *1* and Small Size Map

**Figure 6:**
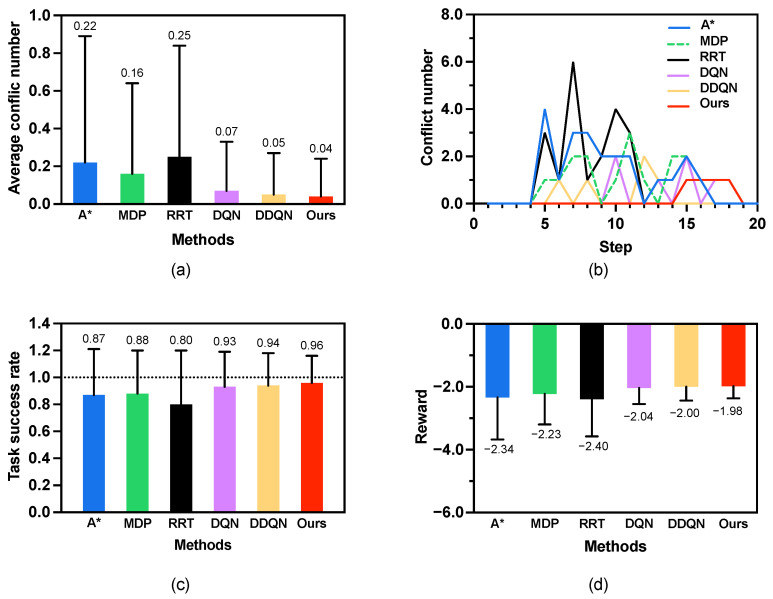
Results of scenario 1: human number K=1 and a 10 × 10 grid map. (**a**) Average conflict number. (**b**) Conflict distribution. (**c**) Average task success rate. (**d**) Average reward.

This experiment was conducted in a 10×10 grid world (Figure 5a) environment to evaluate the performance of the proposed method against three baseline methods: A*, MDP, RRT, DQN, and DDQN. The agent starts from $s_{0}$ = (0, 0), and $s_{g}$ = (9, 9) is its goal position. A human moves stochastically from position $h_{0}^{1}$ = (9, 0) to his/her goal position $h_{g}^{1}$ = (0, 9). The time budget T=20. The results, summarized in Figure 6 and Table 2.

(1) Average conflict number. Figure 6a shows the results for the average number of conflicts. With an average of only 0.04 ± 0.20 conflicts, our proposed method significantly outperforms all baselines: A* (0.22 ± 0.67), MDP (0.16 ± 0.48), RRT (0.25 ± 0.59), DQN (0.07 ± 0.26), and DDQN (0.05 ± 0.22). The low standard deviation further indicates that this performance is consistent and reliable. It shows that our method is much better than A*, MDP and RRT methods; slightly better than DQN and DDQN methods with the small size map.

(2) Conflict distribution. Figure 6b illustrates the time step distribution of conflicts that occurred during the simulations. Our proposed method MP-RRT demonstrates a significantly superior performance, with the first conflict occurring at t=15 far behind other methods. Its distribution is nearly towards zero conflicts. In contrast, the baseline methods (A*, MDP, RRT, DQN, and DDQN) show a much wider spread and a higher frequency of conflicts, which indicate that their strategies are less effective at predicting and avoiding the stochastically moving human. This shows a great advantage of the proposed method in proactively resolving future conflicts. As the time step increases, conflict resolution becomes more challenging due to the inherent uncertainty and dynamic nature of future.

(3) Average task success rate. Figure 6c presents the average task success rate, which measures the agent’s ability to reach its goal within the allotted time budget (T=20). The proposed method achieves the highest success rate of 96% (standard deviation ± 0.20), surpassing the performance of A* (87%), MDP (88%), RRT (80%), DQN (93%), and DDQN (94%). This indicates that our method’s strategy for avoiding conflicts does not come at the cost of failing its primary objective; instead, it more effectively navigates the dynamic environment to reach the goal reliably.

(4) Average reward. Figure 6d depicts the average path reward. Our method achieves the highest average reward of −1.98 ± 0.39, which is notably better than the rewards of A* (−2.34 ± 1.34), MDP (−2.23 ± 0.97), RRT (−2.40 ± 1.18), DQN (−2.04 ± 0.51), and DDQN (−2.00 ± 0.44). The average path reward shows that the path quality of our method is better than the other methods.

The combination of the highest success rate, lowest conflict number, and highest reward confirms that the proposed method offers a more robust and optimal solution for safe path planning in a human-shared space.

### 5.3. Verification on Scenario 2: Different Size Maps

In Scenario 2, we evaluate the performance of the proposed algorithm on larger grid maps of sizes 20×20 and 40×40, as illustrated in Figure 5b,c. This scenario considers only one human in this scenario. The time budgets allocated for the agent are set to T=40 and T=80 for the 20×20 and 40×40 maps, respectively. The simulation results are summarized in Table 2.

**Figure 7:**
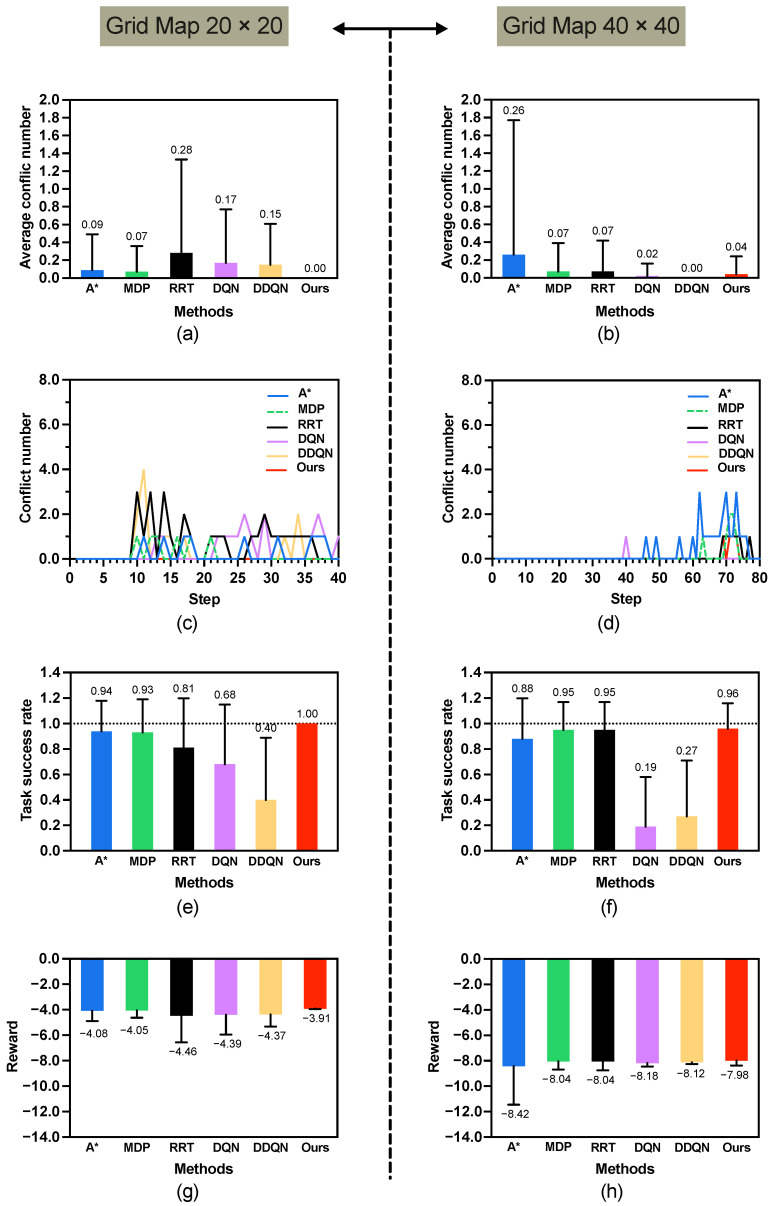
Average results of 20×20 and 40×40 grid map. (**a**,**b**) are conflict number of 20×20 and 40×40 map, respectively. (**c**,**d**) present the conflict distributions. (**e**,**f**) illustrate the task success rates, while (**g**,**h**) depict the rewards for 20×20 and 40×40 map, respectively.

**Figure 8:**
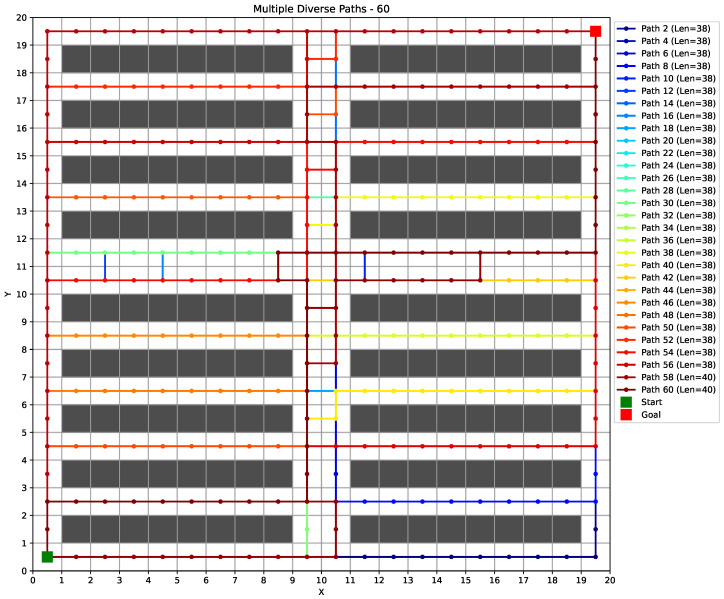
An example of the generated diverse paths on a 20×20 map, where 60 diverse paths are generated. For brevity, only even-numbered paths are displayed in the legend. The “len” in the legend denotes the length of a path.

(1) Results of 20×20 map. As shown in Figure 7 left and Table 2, the proposed method achieves perfect performance in both task success rate (1.00 ± 0.00) and conflict avoidance (0.00 ± 0.00), significantly outperforming all baseline methods across all three metrics. The obtained reward of −3.91 ± 0.04 is also the highest among all approaches, with statistically significant differences observed in every comparative case. This indicates that the method is highly effective in medium-scale environments. Figure 8 illustrates a set of diverse paths generated by our MP-RRT algorithm. As can be seen from the figure, every location on the map has been explored.

(2) Results of 40×40 map. In contrast, on the larger map (Figure 7 right and Table 2), while the proposed method still attains the highest overall reward (−7.98 ± 0.39) and a strong task success rate (0.96 ± 0.20). In terms of conflict number, although the proposed method (0.04 ± 0.20) is slightly higher than DDQN (0.00 ± 0.00), the task success rate of DDQN reduces to a low-level of (0.27 ± 0.44). Similarly, in task success rate, it performs comparably to MDP and RRT, with no significant differences detected. These results suggest that as environmental complexity increases, although the conflict number of our method is not the minimal, the results demonstrate that it achieves the highest task success rate.

Table 2 indicates that our method achieves superior performance on the 20×20 map compared to the 40×40 map. To verify this observation, we changed the human’s start and goal positions and conducted a new simulation, $h_{0}^{1}:\left(30,39\right)\to \left(39,0\right)$, $h_{g}^{1}:\left(15,2\right)\to \left(0,39\right)$. The results are as follows: average task success rate 1.00±0.00, average conflict number 0.00±0.00, and average reward −7.91±0.06. Compared with the results in Table 2, it is difficult to conclude that our algorithm performs better in the 20×20 map. Nevertheless, it can be confirmed that the proposed method performs consistently well in both 20×20 and 40×40 map environments.

Table 2 also shows that DQN and DDQN do not have good performances when map size increases to 20×20 and 40×40. As the grid map size increases, the performance of DQN and DDQN degrades due to the enlarged state space, sparse reward signals, and insufficient exploration, which hinder the convergence of value estimation. In contrast, model-based approaches such as MDP maintain stable performance owing to their explicit transition modeling and global optimization nature.

From Figure 6 and Figure 7, it can be observed that increasing the map size has little impact on our method, which consistently maintains a low number of conflicts and a high task success rate. This robustness arises from the use of the MP-RRT diverse path generator, which computes multiple diverse paths across the entire environment. As a result, our method is able to identify safe paths by considering the global environment rather than being restricted to local regions.

### 5.4. Verification on Scenario 3: Increasing Human Number K

**Figure 9:**
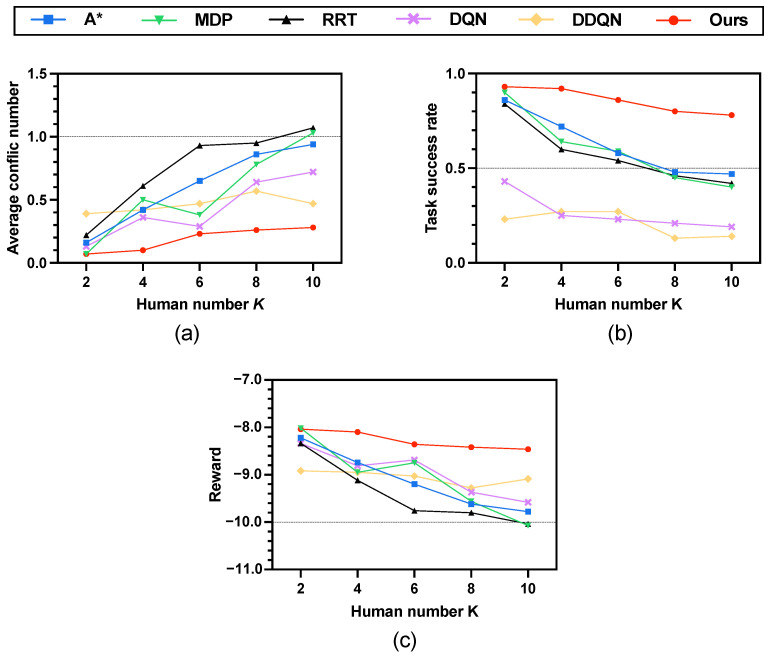
Simulation results on increasing human number *K*. (**a**) Average conflict number. (**b**) Average task success rate. (**c**) Average reward.

In this setting, we evaluate the proposed method in a more challenging environment by increasing the number of humans to K∈{2,4,…,10}. The simulation is a 40 × 40 grid world (Figure 5c), where the agent starts at $s_{0}=\left(0,0\right)$ and aims to reach $s_{g}=\left(39,39\right)$ within a time budget of T=80 steps. Human start and goal locations are randomly initialized; a representative instance uses $h_{0}^{1}$ = (30, 39), $h_{0}^{2}$ = (39, 0), $h_{0}^{3}$ = (4, 2), $h_{0}^{4}$ = (1, 37), $h_{0}^{5}$ = (29, 31), $h_{0}^{6}$ = (2, 33), $h_{0}^{7}$ = (14, 4), $h_{0}^{8}$ = (21, 8), $h_{0}^{9}$ = (30, 10), $h_{0}^{10}$ = (33, 12); with corresponding goals $h_{g}^{1}$ = (15, 2), $h_{g}^{2}$ = (0, 39), $h_{g}^{3}$ = (35, 35), $h_{g}^{4}$ = (32, 6), $h_{g}^{5}$ = (6, 12), $h_{g}^{6}$ = (30, 2), $h_{g}^{7}$ = (38, 29), $h_{g}^{8}$ = (5, 27), $h_{g}^{9}$ = (6, 25), and $h_{g}^{10}$ = (17, 35). The time budget is configured as T=80. Figure 9 illustrates the simulation results across three key metrics, while Table 3 provides a detailed summary.

(1) Average conflict number. As shown in the Figure 9a, all baseline methods exhibit a steady increase in collisions as the human number grows, with RRT suffering the most. In contrast, our method maintains consistently low conflict numbers across all scenarios.

(2) Average task success rate. For the task success rate (Figure 9b, the baselines experience a sharp decline as the environment becomes more crowded. Success rates of A*, MDP, and RRT all drop below 0.5 when K≥8. By comparison, the proposed method sustains a high success rate above 0.8 even in the most challenging case with 10 humans.

(3) Average reward. Figure 9c shows the results of average reward. Our method consistently outperforms the baselines. Although rewards decrease slightly with larger *K*, the reduction is marginal compared with the steep declines observed for A*, MDP, and RRT. This indicates that our approach not only achieves higher success rates but also balances efficiency and safety better than the alternatives.

In Table 3, when the number of humans is K=2, both the MDP method and the proposed method achieve the same average conflict number (0.07±0.26). This mainly results from two factors: (1) the 40×40 map is relatively sparse with only two humans, and (2) both methods adopt similar conflict resolution mechanisms based on soft constraints. The RL method learns an optimal path by penalizing conflicts in the reward function, while our method optimizes the risk level of each path candidate using Equation (14) under simulated risk constraints. With such sparse human distribution and similar mechanisms, both methods yield comparable results. As *K* increases and human-induced risks become stronger, their performance differences become more distinct. As shown in Table 3, the performances of DQN and DDQN remain poor for the same reason as discussed previously, which is consistent with the results in Table 2.

**Figure 10:**
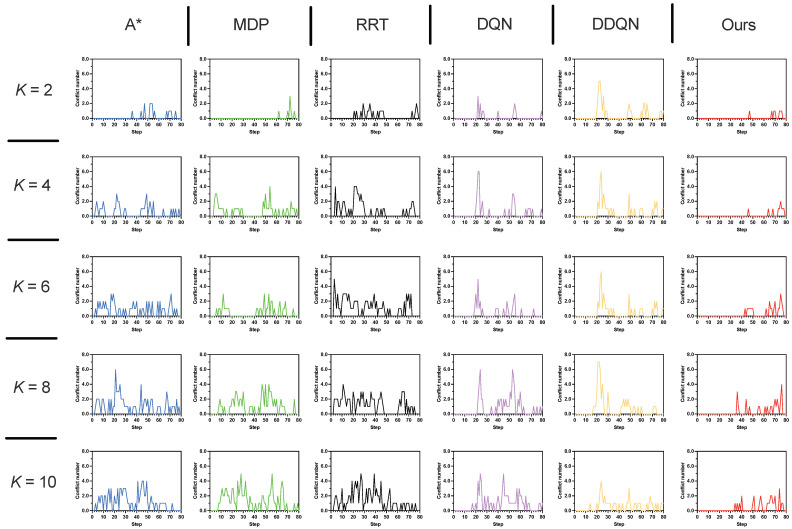
Conflict distribution of a 40×40 grid map with different human number.

Figure 10 illustrates the conflict distribution across varying human numbers *K* ranging from 2 to 10 in a 40×40 grid map. The proposed method consistently demonstrates robust performance as *K* increases, maintaining a notably low conflict frequency across all scenarios. For smaller human numbers (e.g., K=2 and K=4), conflicts are exceptionally rare with the majority of trials resulting in zero conflicts. With *K* increasing to 6 and 8, there is a marginal increase in the occurrence of conflicts. That said, the distribution still exhibits a strong skew toward lower counts—an observation that underscores the effectiveness of conflict avoidance, even as environmental complexity intensifies. Even at K=10, where the environment is most crowded and dynamic, the proposed method reduces conflicts by 70.2%, 72.8%, and 73.8% compared with A*, MDP, and RRT, respectively $\left(\frac{0.94-0.28}{0.94}=70.2\%,\frac{1.03-0.28}{1.03}=72.8\%,\frac{1.07-0.28}{1.07}=73.8\%\right)$. Furthermore, it achieves a significant improvement in the average task success rate, outperforming A* by 66.0%, MDP by 95.0%, and RRT by 85.7% $\left(\frac{0.78-0.47}{0.47}=66.0\%,\frac{0.78-0.40}{0.40}=95.0\%,\frac{0.78-0.42}{0.42}=85.7\%\right)$. This contrast underscores the superiority of the proposed approach in handling scalability and uncertainty in multi-human environments. The results confirm that our proposed method effectively balances path efficiency and safety.

In highly dynamic environments, such as those scenarios involving stochastically moving humans, depth-first search (DFS) methods struggle to handle the pathfinding problem efficiently. In contrast, a key advantage of our approach is the use of a breadth-first search (BFS) scheme to generate multiple diverse path candidates. The proposed multi-policy sampling strategy ensures that these candidates are distributed across the entire map where classic RRT, A*, and RL methods cannot. Thereby, it enhances the ability to cope with dynamic changes. Thus, the algorithm is more robust to the dynamic environment. The safety of the generated path is ensured from the human risks with stochastic evaluation module. Such that we can classify the dangerous areas and find an optimal path. Note that there may be no safe path exists as the humans can move anywhere. In such a case our algorithm return a path with lowest risk Risk(x). That is why a conflict still occurs in the results.

It should be noted that, for safety considerations of both the robot and humans, we conducted only simulation experiments for method validation at the current stage, and the proposed approach has not yet been tested in real-world environments. The simulation results demonstrate the efficiency of our algorithm in resolving conflicts at the planning level. However, it still cannot guarantees no conflict occur with planning only one time. The conflict distribution reveals an interesting observation: in our algorithm, conflicts, when they occur, tend to arise much later compared with traditional approaches. In this paper, our algorithm cannot provide a safety guarantee for the entire path theoretically. We only use soft-constraints and calculate the risk level of each path candidates. An optimal path is obtained by minimizing the risk level with Equation (14). However, it still cannot provide the safety guarantee. It is difficult to guarantee 100% safety for the entire path. To improve our algorithm, we will take hard-constraints to provide a safety guarantee in future. Another solution is to divide the path planning problem into several time-horizon sub-problems to ensure the safety for each time-horizon which is much easier. Dividing the path planning problem into several time-horizon sub-problems may be a compromise approach to balance the safety and efficiency.

A limitation of this work is that the computational cost has not been taken into consideration. At the current stage, our primary focus is on ensuring path safety. Once safety can be reliably guaranteed, our next objective will be to accelerate the search process and reduce computational cost. Therefore, in this stage of our research, ensuring the safety of the generated path in human-shared environments is our foremost priority, while computational efficiency will be addressed in our future work.

## 6. Conclusions

In this work, we addressed the challenge of safe path planning for mobile robots operating in human-shared environments with stochastic human motions. To this end, we proposed a multi-policy rapidly-exploring random tree (MP-RRT)-based safe pathfinding algorithm that explicitly incorporates human-related uncertainties at the planning level. The algorithm integrates three key components: (i) a multi-policy RRT diverse path generator that produces multiple candidate paths with enhanced diversity, (ii) a dynamic quadrant stochastic exploration mechanism that improves search efficiency, and (iii) a safe path optimization mechanism based on stochastic risk evaluation to ensure safety in the presence of unpredictable human movements.

Extensive simulations demonstrated that the proposed MP-RRT significantly reduces conflicts by 70.2%, 72.8%, and 73.8% compared with A*, MDP, and RRT, respectively, while improving task success rates by 66.0%, 95.0%, and 85.7%. In addition, the proposed method is more robust to map size than DQN and DDQN methods. These results confirm the effectiveness and robustness of our approach in generating safe and reliable paths in crowded and dynamic human-shared environments.

Our future work will focus on extending the framework to real-world robotic systems and exploring adaptive mechanisms for handling more complex human behaviors and multi-agent interactions.

## Figures and Tables

**Table 2:** Simulation results on different size of maps with the human number K=1. The asterisk (*) denotes a statistically significant difference from the proposed method (p<0.05, Wilcoxon test), whereas the hyphen (-) indicates no significant difference (p≥0.05).

Grid Map	Methods	Average Conflict Number	Task Success Rate	Reward
10×10	A*	0.22 ± 0.67 (*)	0.87 ± 0.34 (*)	−2.34 ± 1.34 (*)
	MDP	0.16 ± 0.48 (*)	0.88 ± 0.32 (*)	−2.23 ± 0.97 (*)
	RRT	0.25 ± 0.59 (*)	0.80 ± 0.40 (*)	−2.40 ± 1.18 (*)
	DQN	0.07 ± 0.26 (-)	0.93 ± 0.26 (-)	−2.04 ± 0.51 (*)
	DDQN	0.05 ± 0.22 (-)	0.94 ± 0.24 (-)	−2.00 ± 0.44 (*)
	**Ours**	**0.04 ± 0.20**	**0.96 ± 0.20**	**−1.98 ± 0.39**
20×20	A*	0.09 ± 0.40 (*)	0.94 ± 0.24 (*)	−4.08 ± 0.80 (*)
	MDP	0.07 ± 0.29 (*)	0.93 ± 0.26 (*)	−4.05 ± 0.58 (*)
	RRT	0.28 ± 1.05 (*)	0.81 ± 0.39 (*)	−4.46 ± 2.10 (*)
	DQN	0.17 ± 0.60 (*)	0.68 ± 0.47 (*)	−4.39 ± 1.55 (*)
	DDQN	0.15 ± 0.46 (*)	0.40 ± 0.49 (*)	−4.37 ± 0.94 (*)
	**Ours**	**0.00 ± 0.00**	**1.00 ± 0.00**	**−3.91 ± 0.04**
40×40	A*	0.26 ± 1.51 (*)	0.88 ± 0.32 (*)	−8.42 ± 3.03 (*)
	MDP	0.07 ± 0.32 (-)	0.95 ± 0.22 (-)	−8.04 ± 0.65 (-)
	RRT	0.07 ± 0.35 (-)	0.95 ± 0.22 (-)	−8.04 ± 0.71 (-)
	DQN	0.02 ± 0.14 (-)	0.19 ± 0.39 (*)	−8.18 ± 0.27 (*)
	DDQN	**0.00 ± 0.00** (*)	0.27 ± 0.44 (*)	−8.12 ± 0.13 (*)
	**Ours**	0.04 ± 0.20	**0.96 ± 0.20**	**−7.98 ± 0.39**

**Table 3:** Average results under the environmental setting of a 40×40 grid map and 2∼10 humans. The asterisk (*) denotes a statistically significant difference from the proposed method (p<0.05, Wilcoxon test), whereas the hyphen (-) indicates no significant difference (p≥0.05).

Human Number	Methods	Conflict Number	Task Success Rate	Reward
K=2	A*	0.16 ± 0.42 (-)	0.86 ± 0.35 (-)	−8.22 ± 0.84 (-)
	MDP	0.07 ± 0.26 (-)	0.90 ± 0.30 (-)	**−8.02 ± 0.51** (-)
	RRT	0.22 ± 0.60 (*)	0.84 ± 0.37 (*)	−8.34 ± 1.19 (*)
	DQN	0.13 ± 0.36 (*)	0.43 ± 0.50 (*)	−8.34 ± 0.89 (*)
	DDQN	0.39 ± 0.72 (*)	0.23 ± 0.42 (*)	−8.92 ± 1.53 (*)
	**Ours**	**0.07 ± 0.26**	**0.93 ± 0.26**	−8.04 ± 0.51
K=4	A*	0.42 ± 0.89 (*)	0.72 ± 0.45 (*)	−8.74 ± 1.77 (*)
	MDP	0.50 ± 1.22 (*)	0.64 ± 0.48 (*)	−8.95 ± 2.46 (*)
	RRT	0.61 ± 1.18 (*)	0.60 ± 0.49 (*)	−9.12 ± 2.36 (*)
	DQN	0.36 ± 0.54 (*)	0.25 ± 0.43 (*)	−8.81 ± 1.08 (*)
	DDQN	0.42 ± 0.81 (*)	0.27 ± 0.44 (*)	−8.95 ± 1.67 (*)
	**Ours**	**0.10 ± 0.39**	**0.92 ± 0.27**	**−8.10 ± 0.77**
K=6	A*	0.65 ± 1.19 (*)	0.58 ± 0.49 (*)	−9.20 ± 2.39 (*)
	MDP	0.38 ± 0.78 (*)	0.59 ± 0.49 (*)	−8.75 ± 1.56 (*)
	RRT	0.93 ± 1.81 (*)	0.54 ± 0.50 (*)	−9.76 ± 3.61 (*)
	DQN	0.29 ± 0.60 (*)	0.23 ± 0.42 (*)	−8.69 ± 1.21 (*)
	DDQN	0.47 ± 1.20 (*)	0.27 ± 0.44 (*)	−9.03 ± 2.42 (*)
	**Ours**	**0.23 ± 0.76**	**0.86 ± 0.35**	**−8.36 ± 1.52**
K=8	A*	0.86 ± 1.23 (*)	0.48 ± 0.50 (*)	−9.62 ± 2.47 (*)
	MDP	0.78 ± 1.21 (*)	0.45 ± 0.50 (*)	−9.56 ± 2.42 (*)
	RRT	0.95 ± 1.25 (*)	0.46 ± 0.50 (*)	−9.80 ± 2.50 (*)
	DQN	0.64 ± 1.42 (*)	0.21 ± 0.41 (*)	−9.37 ± 2.85 (*)
	DDQN	0.57 ± 0.89 (*)	0.13 ± 0.34 (*)	−9.28 ± 1.79 (*)
	**Ours**	**0.26 ± 0.58**	**0.80 ± 0.40**	**−8.42 ± 1.15**
K=10	A*	0.94 ± 1.29 (*)	0.47 ± 0.50 (*)	−9.78 ± 2.57 (*)
	MDP	1.03 ± 1.84 (*)	0.40 ± 0.49 (*)	−10.07 ± 3.69 (*)
	RRT	1.07 ± 1.44 (*)	0.42 ± 0.50 (*)	−10.04 ± 2.87 (*)
	DQN	0.72 ± 1.50 (*)	0.19 ± 0.39 (*)	−9.58 ± 3.14 (*)
	DDQN	0.47 ± 0.77 (*)	0.14 ± 0.35 (*)	−9.09 ± 1.58 (*)
	**Ours**	**0.28 ± 0.63**	**0.78 ± 0.41**	**−8.46 ± 1.27**

## Data Availability

The data generated by the proposed algorithm are shown in the manuscript and no additional data is used.

## Abbreviations

The following abbreviations are used in this manuscript:

RL	Reinforcement Learning
MDP	Markov Decision Process
RRT	Rapidly-Exploring Random Tree
MP-RRT	Multi-Policy Rapidly Exploring Random Tree
DQN	Deep Q-network
DDQN	Double Deep Q-network
